# Tracing regulatory element networks using epigenetic traits to identify key transcription factors: TENET R/Bioconductor package

**DOI:** 10.1093/bioinformatics/btaf435

**Published:** 2025-08-01

**Authors:** Daniel J Mullen, Zexun Wu, Ethan Nelson-Moore, Huan Cao, Lauren Han, Ite A Offringa, Suhn K Rhie

**Affiliations:** Department of Cancer Biology, Norris Comprehensive Cancer Center, Keck School of Medicine, University of Southern California, Los Angeles, CA 90033, United States; Department of Surgery, Keck School of Medicine, University of Southern California, Los Angeles, CA 90033, United States; Department of Cancer Biology, Norris Comprehensive Cancer Center, Keck School of Medicine, University of Southern California, Los Angeles, CA 90033, United States; Department of Cancer Biology, Norris Comprehensive Cancer Center, Keck School of Medicine, University of Southern California, Los Angeles, CA 90033, United States; Department of Cancer Biology, Norris Comprehensive Cancer Center, Keck School of Medicine, University of Southern California, Los Angeles, CA 90033, United States; Department of Cancer Biology, Norris Comprehensive Cancer Center, Keck School of Medicine, University of Southern California, Los Angeles, CA 90033, United States; Department of Cancer Biology, Norris Comprehensive Cancer Center, Keck School of Medicine, University of Southern California, Los Angeles, CA 90033, United States; Department of Surgery, Keck School of Medicine, University of Southern California, Los Angeles, CA 90033, United States; Department of Cancer Biology, Norris Comprehensive Cancer Center, Keck School of Medicine, University of Southern California, Los Angeles, CA 90033, United States

## Abstract

**Summary:**

There is a lack of publicly available bioinformatic tools that can be widely used by researchers to identify transcription factors (TFs) that regulate cell type-specific regulatory elements (REs). To address this, we developed the Tracing regulatory Element Networks using Epigenetic Traits (TENET) R/Bioconductor package. By collecting hundreds of histone mark and open chromatin datasets from a variety of cell lines, primary cells, and tissues, and comparing these features along with matched DNA methylation and gene expression data, TENET identifies TFs and REs linked to a specific cell type. Moreover, we developed methods to interrogate findings using motifs, clinical information, and other genomic and chromatin conformation capture datasets, and applied them to pan-cancer data, highlighting TFs and REs associated with ten different cancer types. TENET enables researchers to better characterize the 3D epigenomes of cell types of interest for future clinical applications.

**Availability and implementation:**

TENET is available at http://bioconductor.org/packages/TENET. Curated functional genomic datasets utilized by TENET are available at http://bioconductor.org/packages/TENET.AnnotationHub. Example datasets are available at http://bioconductor.org/packages/TENET.ExperimentHub.

## 1 Introduction

Transcription factors (TFs) bind to regulatory elements (REs) to control the expression of numerous genes across the genome. REs include promoters, which are located proximally to the transcription start sites (TSSs) of the genes they regulate, and enhancers, which are located at a distance from the TSSs of their target genes. The locations of REs in a given cell type can be ascertained by profiling histone marks, including histone 3 lysine 4 trimethylation (H3K4me3, a mark of active promoters), histone 3 lysine 4 monomethylation (H3K4me1, a mark of poised and active enhancers), and histone 3 lysine 27 acetylation (H3K27ac, a mark of active enhancers) using ChIP-seq and its derivative methods such as CUT&RUN and CUT&Tag. DNase-seq and ATAC-seq can identify nucleosome-depleted regions (NDRs), which are open chromatin regions where TFs bind within REs. However, assessing the activities of REs in some cell and tissue types using these techniques remains challenging, as they necessitate the use of a substantial number of cells, are time-consuming to perform, and may be hindered by difficulties in acquiring fresh tissues and intact nuclei (Lee and Rhie 2021).

DNA methylation is one of the most thoroughly studied forms of epigenetic modification. DNA methylation levels near REs correlate inversely with the activities of those elements (Stadler *et al.* 2011, Aran *et al.* 2013, Blattler and Farnham 2013), indicating that DNA methylation can be utilized to annotate specific REs where TFs bind. DNA methylation is easily assayed from any type of cell and tissue, including formalin-fixed, paraffin-embedded (FFPE) tissue samples, using very few cells (Hinoue *et al.* 2012, Farlik *et al.* 2015).

Several studies have utilized DNA methylation and RNA-seq data to identify REs and their target genes in different cell types (Yao *et al.* 2015, Heyn *et al.* 2016, Rhie *et al.* 2016, Fleischer *et al.* 2017, Silva *et al.* 2019, Detilleux *et al.* 2022). However, a deficiency exists in publicly available and easily accessible bioinformatic tools for identifying TFs that regulate REs from DNA methylation and RNA-seq data by incorporating histone mark and open chromatin datasets. To address this gap, we upgraded previous TENET frameworks (Rhie *et al.* 2016, Mullen *et al.* 2020) and developed an R/Bioconductor package, which incorporates DNA methylation datasets, as well as datasets of histone marks and open chromatin, to identify and assess the activities of REs (promoters and enhancers). The TENET R/Bioconductor package also includes algorithms to allow users to easily combine epigenomic datasets with RNA-seq datasets to identify important TFs linked to RE dysregulation, which drive individual subgroups of cases compared to controls. Furthermore, it has numerous functions to interrogate findings with TF motif databases, patient survival information, and other genomic and chromatin conformation capture datasets. To demonstrate the effectiveness of the TENET R/Bioconductor package, we applied it to perform a pan-cancer analysis on datasets from ten distinct cancer types. These insights provide valuable information for understanding gene regulation in various cell types, as well as identifying potential biomarkers and therapeutic targets for clinical intervention.

## 2 Feature highlights

### 2.1 TENET design and features

**Figure 1.**
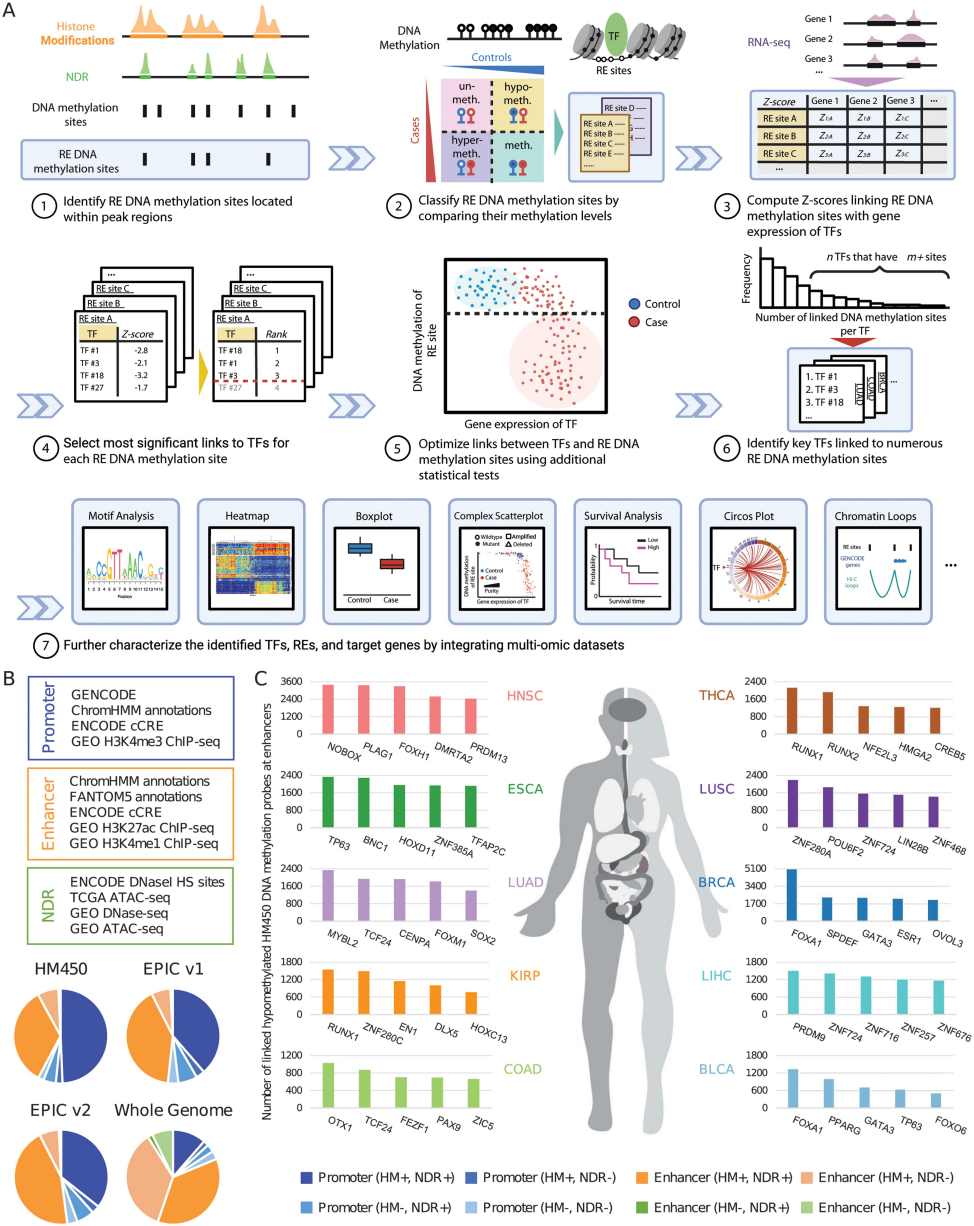
Overview of the TENET R/Bioconductor package. (A) TENET includes seven steps. In step 1, histone mark and open chromatin data that annotate regulatory elements (REs) and nucleosome-depleted regions (NDRs) are used to identify DNA methylation sites which are located in REs. In step 2, using DNA methylation data, RE DNA methylation sites are classified into four different categories based on their methylation levels in case and control samples: unmethylated in both, hypomethylated in case compared to control, hypermethylated in case compared to control, and methylated in both. In step 3, using matched gene expression data, TENET computes Z-scores linking RE DNA methylation sites with gene expression to identify the transcription factor (TF) gene-RE site links genome-wide which show significant differences in DNA methylation of the RE sites and gene expression in a subset of the case samples compared to the control samples. In step 4, statistically significant TF gene–RE site links are identified for each RE DNA methylation site by ranking and performing multiple testing correction on the total number of links. In step 5, by performing additional statistical tests (e.g. Wilcoxon rank-sum test, adjusted *P*-value < .05), TENET optimizes the identification of links. In step 6, by calculating the number of linked RE DNA methylation sites per TF, key TFs linked to numerous RE sites are identified. Finally, step 7 functions perform downstream analyses by integrating multi-omic datasets to better characterize the identified TFs, REs, and their target genes. (B) TENET includes built-in epigenomic datasets to aid the user in identifying DNA methylation sites located in REs. These datasets provide significant coverage of both DNA methylation probes included in Illumina Human Methylation arrays (HM450, EPIC v1, EPIC v2), as well as all CpG sites throughout the genome. (C) Bar plots display the key TFs identified through a pan-cancer TENET analysis using TCGA data. The top 5 TFs linked to the largest number of hypomethylated enhancer sites (HM450 probes) are shown for each cancer type.

The rationale behind TENET is that overexpression of a TF gene in a particular cell type (case) compared to another type (control) can lead to increased binding of the translated TF protein to numerous cell type-specific REs, resulting in widespread changes in the expression of downstream target genes controlled by these REs. Thus, the activities of TFs have important effects on the transcriptome of a cell type, determining cell fate (Fig. 1, available as supplementary data at *Bioinformatics* online). Unlike previous TENET frameworks, the TENET R/Bioconductor package (Fig. 1A) has been newly updated to utilize GenomicRanges and MultiAssayExperiment objects, allowing users to utilize a much wider range of epigenomic data as well as matched DNA methylation and gene expression data. In addition to the main TENET package, we developed the TENET.AnnotationHub package, which contains datasets created by processing hundreds of epigenomic datasets, such as ChIP-seq and open chromatin datasets across cell and tissue types and ten cancer types to allow users to perform analyses without needing their own such datasets representing REs (Fig. 1B, Fig. 2, available as supplementary data at *Bioinformatics* online) (Thurman *et al*. 2012, Andersson *et al*. 2014, Forrest *et al*. 2014, Kundaje *et al*. 2015, Corces *et al.* 2018, Moore *et al*. 2020). We also created the TENET.ExperimentHub package with example datasets for easy use in the TENET R/Bioconductor package. We have added functionality to analyze REs at both promoters and enhancers and have included new algorithms to automatically set methylation cutoffs to identify dysregulated REs (Fig. 3, available as supplementary data at *Bioinformatics* online) in TENET. The package also allows users to interrogate findings with motif searching, perform survival analyses using both the gene expression of key TF genes identified by TENET (Fig. 4, available as supplementary data at *Bioinformatics* online) and the DNA methylation levels of the identified RE sites, generate heatmaps contrasting the expression of the identified TF genes with the DNA methylation of their linked RE sites, and integrate other genomic and chromatin conformation capture datasets.

The TENET R/Bioconductor package currently consists of a collection of functions divided into seven steps that are designed to be run in succession, although a subset of these functions can be used independently if desired (Fig. 1A). The easyTENET wrapper function has also been included, which runs steps 1 through 6 as a single function with simplified options for ease of use.

### 2.2 Regulatory elements and transcription factors linked to 10 cancer types are identified by using TENET

To illustrate the efficacy of using the TENET R/Bioconductor package, we applied it to a panel of Illumina HumanMethylation450 (HM450) DNA methylation and gene expression datasets consisting of both tumor and adjacent-normal samples from ten cancer types, downloaded from The Cancer Genome Atlas (TCGA) (Colaprico *et al.* 2016). Using the integrated epigenomic datasets built into TENET, we identified over 180 000 HM450 DNA methylation probes in promoters and over 90 000 probes in enhancers across the ten cancer types, including bladder urothelial carcinoma (BLCA), breast invasive carcinoma (BRCA), colon adenocarcinoma (COAD), esophageal carcinoma (ESCA), head and neck squamous cell carcinoma (HNSC), kidney renal papillary cell carcinoma (KIRP), liver hepatocellular carcinoma (LIHC), lung adenocarcinoma (LUAD), lung squamous cell carcinoma (LUSC), and thyroid carcinoma (THCA). Of these, the majority of enhancer probes in each cancer type were hypomethylated in tumor (case) compared to normal (control) samples (Fig. 5, available as supplementary data at *Bioinformatics* online), while the majority of promoter probes were hypermethylated.

Next, we identified key TFs whose overexpression is associated with the activation of numerous cell type-specific REs (hypomethylated RE sites) for the ten cancer types (Fig. 1C, Fig. 6, available as supplementary data at *Bioinformatics* online). We found both well-known TFs, reported to be activated in tumors and involved in RE networks, as well as novel TFs. For example, TP63 was one of the key TFs identified to be linked to enhancers in ESCA and BLCA. TP63 has been previously shown to promote the growth of ESCA cells by regulating the cell cycle and the Akt pathway (Ye *et al.* 2014). TP63 has also been previously associated with the basal subtype of BLCA (Iyyanki *et al.* 2021). FOXA1, known as a pioneering TF (Cirillo *et al.* 2002, Iwafuchi-Doi *et al.* 2016, Mayran and Drouin 2018), was identified as the top TF in both BLCA and BRCA. FOXA1 plays a key role in cancer by regulating the nuclear steroid receptors to control the transcriptomes of subtypes of BRCA and BLCA, as highlighted by previous studies (DeGraff *et al.* 2012, Bernardo *et al.* 2013, Rhie *et al.* 2016, Warrick *et al.* 2016, Fu *et al.* 2019, Sikic *et al.* 2020, Iyyanki *et al.* 2021, Seachrist *et al.* 2021). We also identified novel TFs such as NOBOX in HNSC, ZNF280C in KIRP, and POU6F2 in LUSC, which potentially regulate cancer-specific regulatory networks yet remain relatively understudied in those cancer types (Fig. 1C). To further illustrate TENET’s features, we examined the association between the expression of the identified TFs and DNA methylation of their linked RE sites with patient survival, identifying those that are statistically significantly associated with KIRP patient survival (Fig. 7A and B, available as supplementary data at *Bioinformatics* online). Lastly, we identified the location of TF motifs at REs and the potential target genes of REs by integrating ChIP-seq and chromatin conformation capture (e.g. Hi-C) datasets in KIRP and LIHC, respectively (Fig. 7C and D, available as supplementary data at *Bioinformatics* online).

## 3 Conclusions and future directions

The TENET R/Bioconductor package enables the identification of key TFs and REs in the cell type of interest by combining multi-omic datasets. This R/Bioconductor package is a substantially upgraded version of previous TENET frameworks (Rhie *et al.* 2016, Mullen *et al.* 2020), which includes new features and datasets. In this study, we demonstrated the use of TENET to detect cell type-specific TFs and REs that are dysregulated in ten cancer types, highlighting those linked to numerous cancer-specific epigenomic changes. Identified TFs and REs, including ones associated with patient survival, will accelerate the further development of biomarkers and therapeutic strategies. While we used cancer datasets to showcase our method, TENET can utilize DNA methylation and gene expression datasets from any cell or disease group to identify key TFs and REs. All of TENET’s functions, including those for searching TF motifs, using topologically associating domains (TADs) to further characterize the target genes of REs and TFs, and identifying activated or inactivated TFs and REs, serve as valuable resource tools. The supplementary TENET.AnnotationHub package also includes datasets useful for identifying REs, which we created by archiving and processing hundreds of epigenomic datasets.

TENET uses DNA methylation to assess the activity of REs, so we cannot evaluate REs that do not have DNA methylation sites nearby. Although we applied TENET to ten cancer datasets generated from bulk tissues, we anticipate that this approach will be applicable to single-cell methyl-seq and single-cell RNA-seq when sufficient data become available. TFs, REs, and links identified using TENET provide invaluable resources, but functional assays, such as performing TF ChIP-seq and its derivative methods, are still required to validate the predictions, since these links were identified through the evaluation of statistical associations and thus, may include indirectly associated TFs.

## Supplementary Material

btaf435_Supplementary_Data

## Data Availability

The data underlying this article are available in the article and in its online supplementary material.

## References

[btaf435-B1] Andersson R , Gebhard C, Miguel-Escalada I et al An atlas of active enhancers across human cell types and tissues. Nature 2014;507:455–61. 24670763 10.1038/nature12787 PMC5215096

[btaf435-B2] Aran D , Sabato S, Hellman A. DNA methylation of distal regulatory sites characterizes dysregulation of cancer genes. Genome Biol 2013;14:R21. 10.1186/gb-2013-14-3-r21 23497655 PMC4053839

[btaf435-B3] Bernardo GM , Bebek G, Ginther CL et al FOXA1 represses the molecular phenotype of basal breast cancer cells. Oncogene 2013;32:554–63. 22391567 10.1038/onc.2012.62 PMC3371315

[btaf435-B4] Blattler A , Farnham PJ. Cross-talk between site-specific transcription factors and DNA methylation states. J Biol Chem 2013;288:34287–94. 24151070 10.1074/jbc.R113.512517 PMC3843044

[btaf435-B5] Cirillo LA , Lin FR, Cuesta I et al Opening of compacted chromatin by early developmental transcription factors HNF3 (FoxA) and GATA-4. Mol Cell 2002;9:279–89. 11864602 10.1016/s1097-2765(02)00459-8

[btaf435-B6] Colaprico A , Silva TC, Olsen C et al TCGAbiolinks: an R/Bioconductor package for integrative analysis of TCGA data. Nucleic Acids Res 2016;44:e71. 26704973 10.1093/nar/gkv1507 PMC4856967

[btaf435-B7] Corces MR , Granja JM, Shams S et al; Cancer Genome Atlas Analysis Network. The chromatin accessibility landscape of primary human cancers. Science 2018;362:eaav1898. 30361341 10.1126/science.aav1898 PMC6408149

[btaf435-B8] DeGraff DJ , Clark PE, Cates JM et al Loss of the urothelial differentiation marker FOXA1 is associated with high grade, late stage bladder cancer and increased tumor proliferation. PLoS One 2012;7:e36669. 22590586 10.1371/journal.pone.0036669 PMC3349679

[btaf435-B9] Detilleux D , Spill YG, Balaramane D et al Pan-cancer predictions of transcription factors mediating aberrant DNA methylation. Epigenet Chromatin 2022;15:10. 10.1186/s13072-022-00443-w PMC8944071 35331302

[btaf435-B10] Farlik M , Sheffield NC, Nuzzo A et al Single-cell DNA methylome sequencing and bioinformatic inference of epigenomic cell-state dynamics. Cell Rep 2015;10:1386–97. 25732828 10.1016/j.celrep.2015.02.001 PMC4542311

[btaf435-B11] Fleischer T , Tekpli X, Mathelier A et al; Oslo Breast Cancer Research Consortium (OSBREAC). DNA methylation at enhancers identifies distinct breast cancer lineages. Nat Commun 2017;8:1379. 29123100 10.1038/s41467-017-00510-x PMC5680222

[btaf435-B12] Forrest ARR , Kawaji H, Rehli M et al; FANTOM Consortium and the RIKEN PMI and CLST (DGT). A promoter-level mammalian expression atlas. Nature 2014;507:462–70. 24670764 10.1038/nature13182 PMC4529748

[btaf435-B13] Fu X , Pereira R, De Angelis C et al FOXA1 upregulation promotes enhancer and transcriptional reprogramming in endocrine-resistant breast cancer. Proc Natl Acad Sci USA 2019;116:26823–34. 31826955 10.1073/pnas.1911584116 PMC6936436

[btaf435-B14] Heyn H , Vidal E, Ferreira HJ et al Epigenomic analysis detects aberrant super-enhancer DNA methylation in human cancer. Genome Biol 2016;17:11. 26813288 10.1186/s13059-016-0879-2 PMC4728783

[btaf435-B15] Hinoue T , Weisenberger DJ, Lange CPE et al Genome-scale analysis of aberrant DNA methylation in colorectal cancer. Genome Res 2012;22:271–82. 21659424 10.1101/gr.117523.110 PMC3266034

[btaf435-B16] Iwafuchi-Doi M , Donahue G, Kakumanu A et al The pioneer transcription factor FoxA maintains an accessible nucleosome configuration at enhancers for tissue-specific gene activation. Mol Cell 2016;62:79–91. 27058788 10.1016/j.molcel.2016.03.001 PMC4826471

[btaf435-B17] Iyyanki T , Zhang B, Wang Q et al Subtype-associated epigenomic landscape and 3D genome structure in bladder cancer. Genome Biol 2021;22:105. 33858483 10.1186/s13059-021-02325-y PMC8048365

[btaf435-B18] Kundaje A , Meuleman W, Ernst J et al; Roadmap Epigenomics Consortium. Integrative analysis of 111 reference human epigenomes. Nature 2015;518:317–30. 25693563 10.1038/nature14248 PMC4530010

[btaf435-B19] Lee BH , Rhie SK. Molecular and computational approaches to map regulatory elements in 3D chromatin structure. Epigenet Chromatin 2021;14:14. 10.1186/s13072-021-00390-y PMC7980343 33741028

[btaf435-B20] Mayran A , Drouin J. Pioneer transcription factors shape the epigenetic landscape. J Biol Chem 2018;293:13795–804. 29507097 10.1074/jbc.R117.001232 PMC6130937

[btaf435-B21] Moore JE , Purcaro MJ, Pratt HE et al; ENCODE Project Consortium. Expanded encyclopaedias of DNA elements in the human and mouse genomes. Nature 2020;583:699–710. 32728249 10.1038/s41586-020-2493-4 PMC7410828

[btaf435-B22] Mullen DJ , Yan C, Kang DS et al TENET 2.0: identification of key transcriptional regulators and enhancers in lung adenocarcinoma. PLoS Genet 2020; 16: e1009023. 32925947 10.1371/journal.pgen.1009023 PMC7515200

[btaf435-B23] Rhie SK , Guo Y, Tak YG et al Identification of activated enhancers and linked transcription factors in breast, prostate, and kidney tumors by tracing enhancer networks using epigenetic traits. Epigenet Chromatin 2016;9:50. 10.1186/s13072-016-0102-4 PMC5103450 27833659

[btaf435-B24] Seachrist DD , Anstine LJ, Keri RA. FOXA1: a pioneer of nuclear receptor action in breast cancer. Cancers (Basel) 2021;13:5205. 34680352 10.3390/cancers13205205 PMC8533709

[btaf435-B25] Sikic D , Eckstein M, Wirtz RM et al FOXA1 gene expression for defining molecular subtypes of muscle-invasive bladder cancer after radical cystectomy. J Clin Med 2020;9:994. 32252315 10.3390/jcm9040994 PMC7230662

[btaf435-B26] Silva TC , Coetzee SG, Gull N et al ELMER v.2: an R/Bioconductor package to reconstruct gene regulatory networks from DNA methylation and transcriptome profiles. Bioinformatics 2019;35:1974–7. 30364927 10.1093/bioinformatics/bty902 PMC6546131

[btaf435-B27] Stadler MB , Murr R, Burger L et al DNA-binding factors shape the mouse methylome at distal regulatory regions. Nature 2011;480:490–5. 22170606 10.1038/nature10716

[btaf435-B28] Thurman RE , Rynes E, Humbert R et al The accessible chromatin landscape of the human genome. Nature 2012;489:75–82. 22955617 10.1038/nature11232 PMC3721348

[btaf435-B29] Warrick JI , Walter V, Yamashita H et al FOXA1, GATA3 and PPARɣ cooperate to drive luminal subtype in bladder cancer: a molecular analysis of established human cell lines. Sci Rep 2016;6:38531. 27924948 10.1038/srep38531 PMC5141480

[btaf435-B30] Yao L , Shen H, Laird PW et al Inferring regulatory element landscapes and transcription factor networks from cancer methylomes. Genome Biol 2015;16:105. 25994056 10.1186/s13059-015-0668-3 PMC4460959

[btaf435-B31] Ye S , Lee KB, Park MH et al p63 regulates growth of esophageal squamous carcinoma cells via the Akt signaling pathway. Int J Oncol 2014;44:2153–9. 24718831 10.3892/ijo.2014.2374

