# Supplementary material for: Tracing regulatory element networks using epigenetic traits to identify key transcription factors: TENET R/Bioconductor package
Source: Bioinformatics. 2025 Aug 1;41(8):btaf435. doi: 10.1093/bioinformatics/btaf435 (PMC12349384; doi:10.1093/bioinformatics/btaf435)
Supplement: btaf435_Supplementary_Data [file btaf435_supplementary_data.docx]

**Supplementary Figures**

**Supplementary Figure S1. TENET identifies dysregulated TFs and their downstream targeted REs and genes**

TENET is designed to identify key transcription factors (TFs) that are linked to cell type-specific regulatory elements (REs) they bind to, resulting in widespread changes in the expression of downstream target genes, by comparing case and control samples. Specifically, this diagram demonstrates TENET’s rationale for finding overexpressed and activated TFs (left) that bind to numerous activated enhancers and promoters (right) which upregulate downstream target genes in case samples compared to control samples.

**
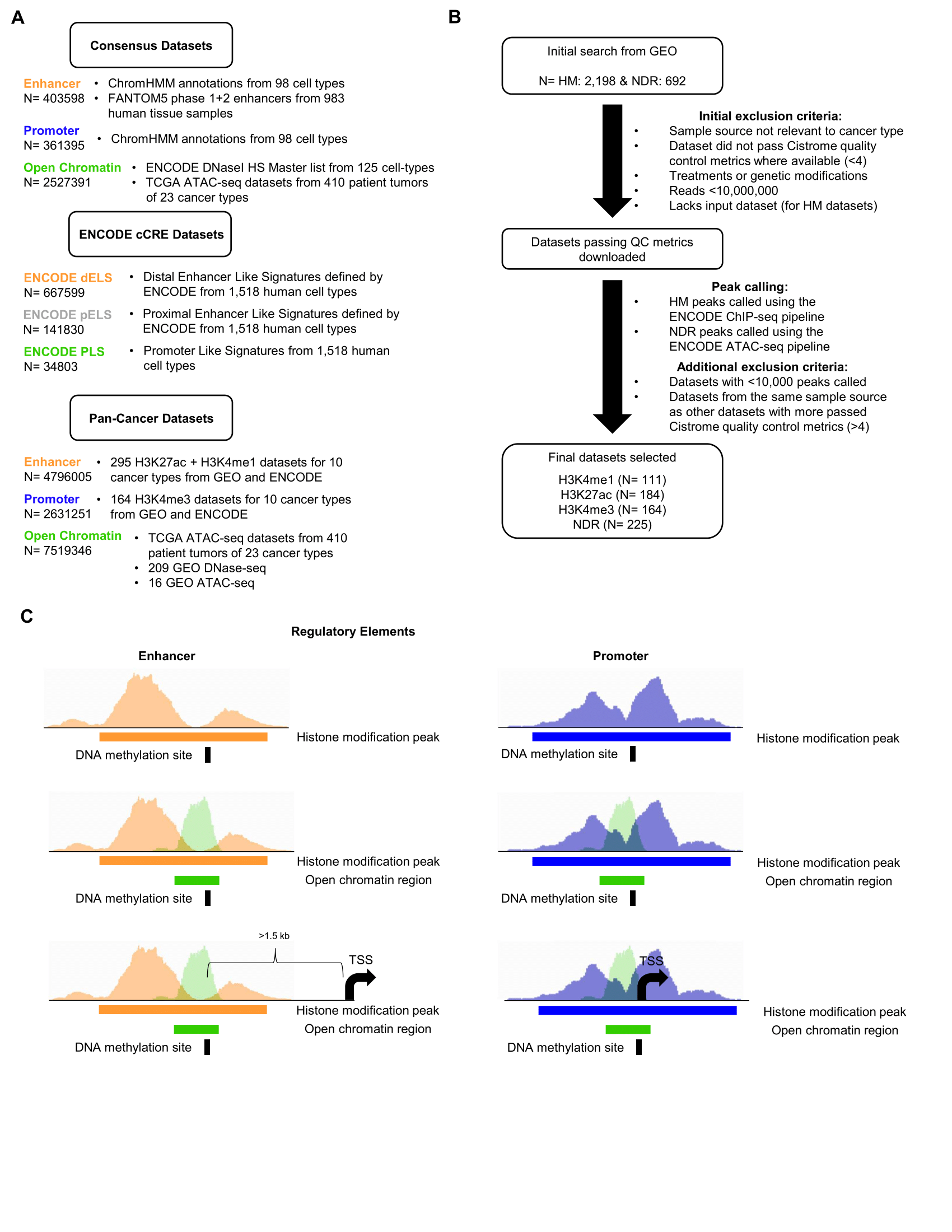
Supplementary Figure S2. Acquisition and processing of epigenomic datasets**

(A) TENET includes 3 epigenomic databases which were built into the TENET.AnnotationHub R/Bioconductor package to aid in the identification of RE DNA methylation sites. The first database is the consensus datasets, which encompass enhancer, promoter, and open chromatin datasets derived from a wide variety of human cell line, tissue, and patient tumor samples from sources such as the ROADMAP Epigenomics ChromHMM annotations, FANTOM5 annotated enhancer regions, ENCODE DNaseI hypersensitive sites Master list, and TCGA ATAC-seq peaks from tumor samples. The second database is the ENCODE cCRE datasets, which represent distal enhancer-like signatures (dELS), proximal enhancer-like signatures (pELS), and promoter-like signatures (PLS) defined as part of Phase III of ENCODE using 1,518 human cell types. The third database is the pan-cancer datasets, for which we processed publicly available raw next-generation sequencing datasets from GEO, ENCODE, and TCGA, representing enhancer, promoter, and open chromatin regions relevant to the ten cancer types of interest in this study. (B) To create the pan-cancer datasets, an initial search was performed using GEO to identify relevant epigenomic datasets by searching for datasets generated from human organs, tissues, and cells of origin for each cancer type. Initial datasets were filtered by removing those which were derived from samples irrelevant to the given cancer type, passed less than four of the quality control metrics (sequence quality, mapping quality, library complexity, ChIP enrichment, signal to noise ratio, and regulatory region) defined by Cistrome (http://cistrome.org/), received an additional chemical treatment or were genetically altered, had less than 10 million reads, or lacked input datasets (for histone mark (HM) datasets only). Raw next-generation sequencing datasets (i.e. FASTQ files) of the samples passing the initial quality control were downloaded and processed to call peaks using the respective pipelines as outlined by ENCODE (https://github.com/ENCODE-DCC). After peak calling, to further remove low-quality datasets, datasets with fewer than 10 thousand peaks called were removed along with any datasets which passed fewer Cistrome quality metrics than another dataset from the same source in the remaining pool of datasets. (C) Diagram of how DNA methylation sites marking REs are identified by TENET. First, DNA methylation sites which overlap histone modification peaks of interest are identified. Next, TENET finds the subset of these sites which also overlap with open chromatin regions (also known as nucleosome-depleted regions (NDRs)). Finally, these sites located outside of (for enhancers) or within (for promoters) a specific distance from annotated transcription start sites (TSSs) are identified as RE DNA methylation sites.

**
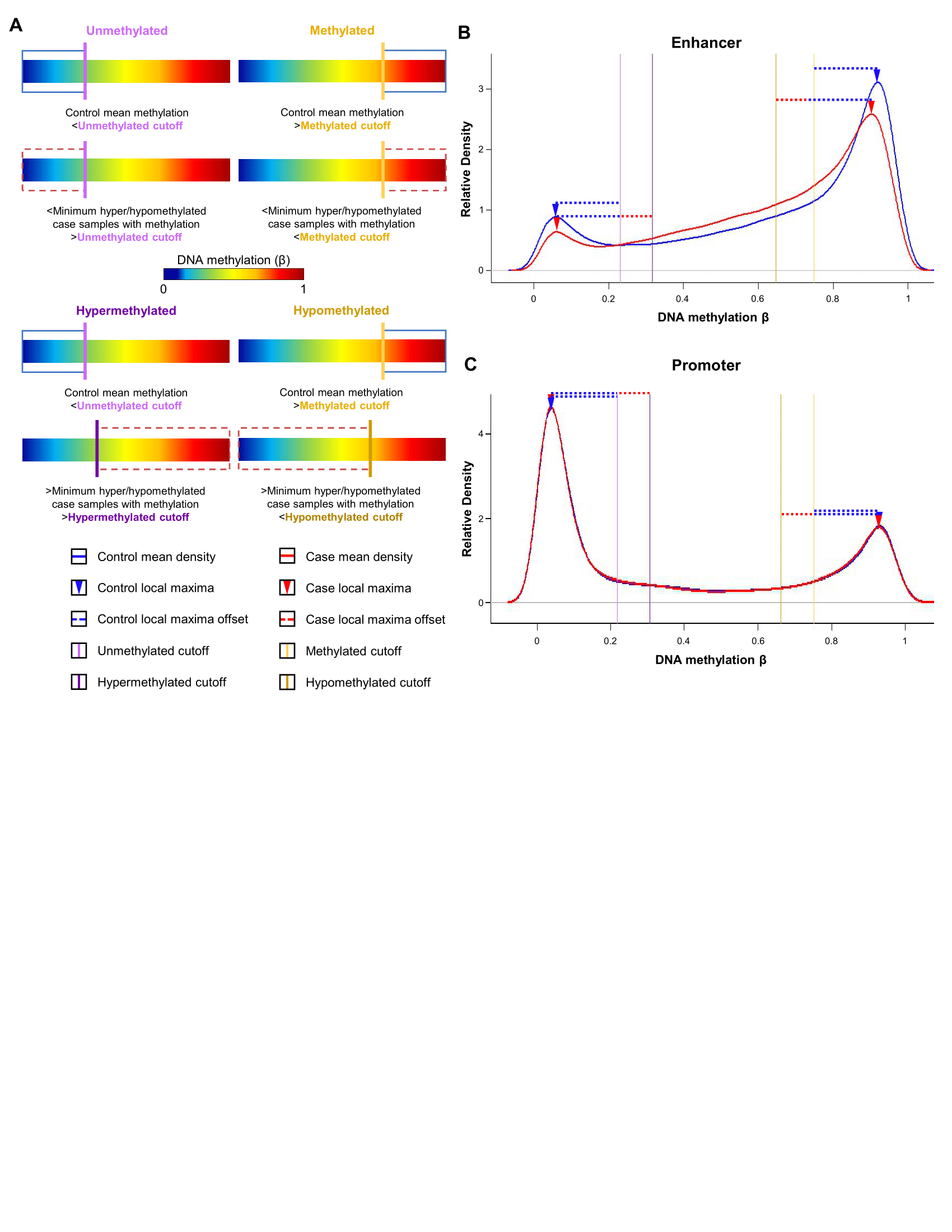
Supplementary Figure S3. Identification of differentially active REs**

(A) RE DNA methylation sites are classified into 4 categories based on DNA methylation levels in case *versus* control samples. Unmethylated and methylated RE sites are those with consistently low and high levels of methylation, representing constitutively active or inactive REs, respectively, in both case and control samples. Hypermethylated sites possess relatively low levels of methylation in control samples and higher levels in the case samples, representing REs that are inactivated in the case samples. In comparison, hypomethylated sites have lower levels of methylation in the case compared to control samples, here representing REs which are activated in the case samples. Density plots of DNA methylation β values of HM450 probes located in (B) enhancers and (C) promoters. TCGA LUAD DNA methylation data was used for these plots. The DNA methylation cutoff values can be defined by users or determined using an algorithm, which automatically sets cutoff values based upon the position of the two local maxima in the bimodal distribution of DNA methylation density of RE sites in case and control samples and the distances between them.

**
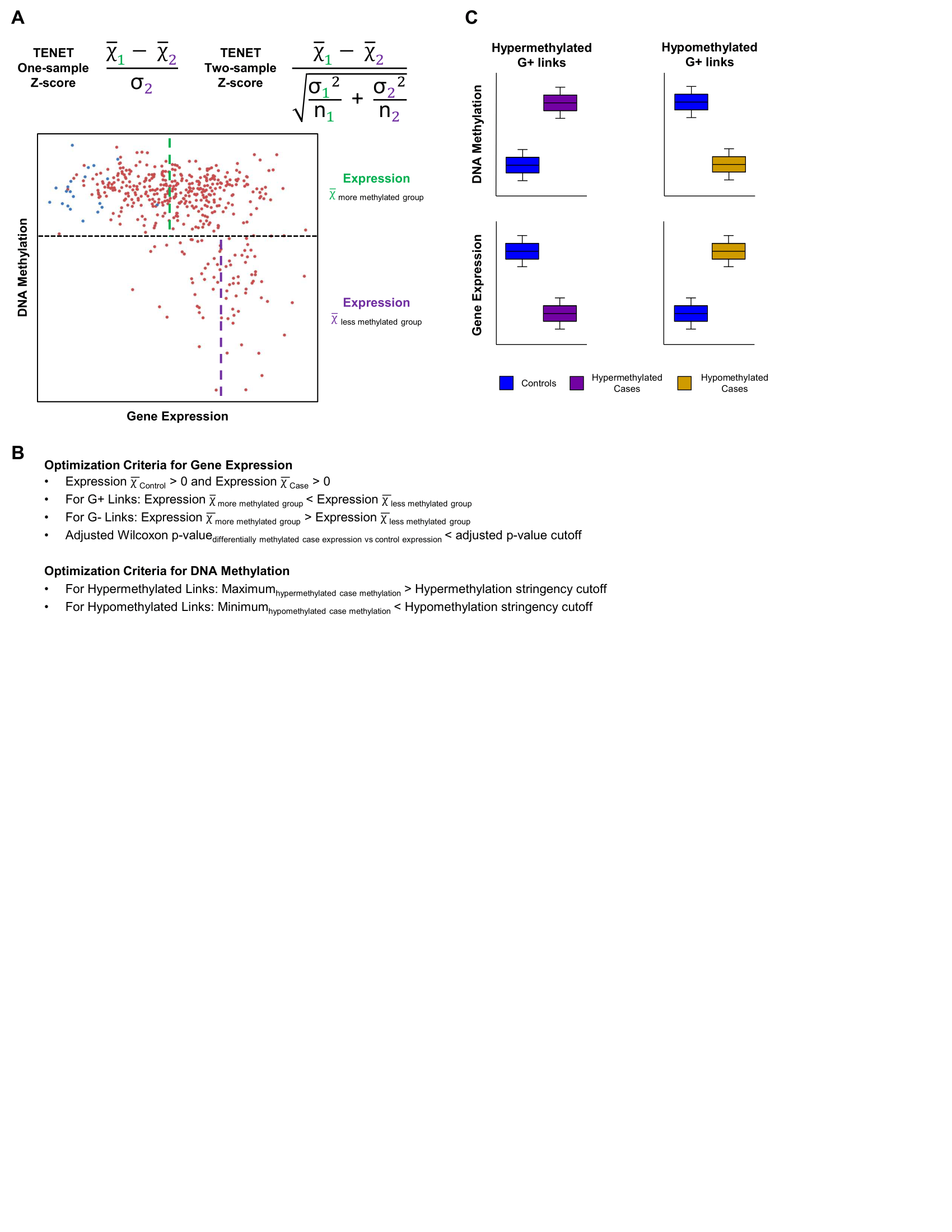
Supplementary Figure S4. TENET identifies TF gene-RE site links using matched DNA methylation and gene expression data**

(A) Formulas for the calculation of Z-scores used in the step3GetAnalysisZScores function along with an illustrated description. For a hypomethylated RE site linked to increased gene expression (Hypomethylated G+ link) as an example, the more methylated group represents samples (including the control (blue) samples) with relatively higher DNA methylation levels of the RE site of interest and lower gene expression levels compared to case (red) samples with lower levels of DNA methylation and higher gene expression levels. (B) List of the statistical criteria the step5OptimizeLinks function uses to select robust TF gene–­­RE site links identified by the step 3 and step 4 functions. (C) Display of expected DNA methylation and gene expression patterns for identified links between significant RE sites and genes. This study mainly reports Hypomethylated G+ links, but TENET can also assess Hypermethylated G+ links consisting of hypermethylated RE sites associated with lower expression of TF genes in cases than controls.

**
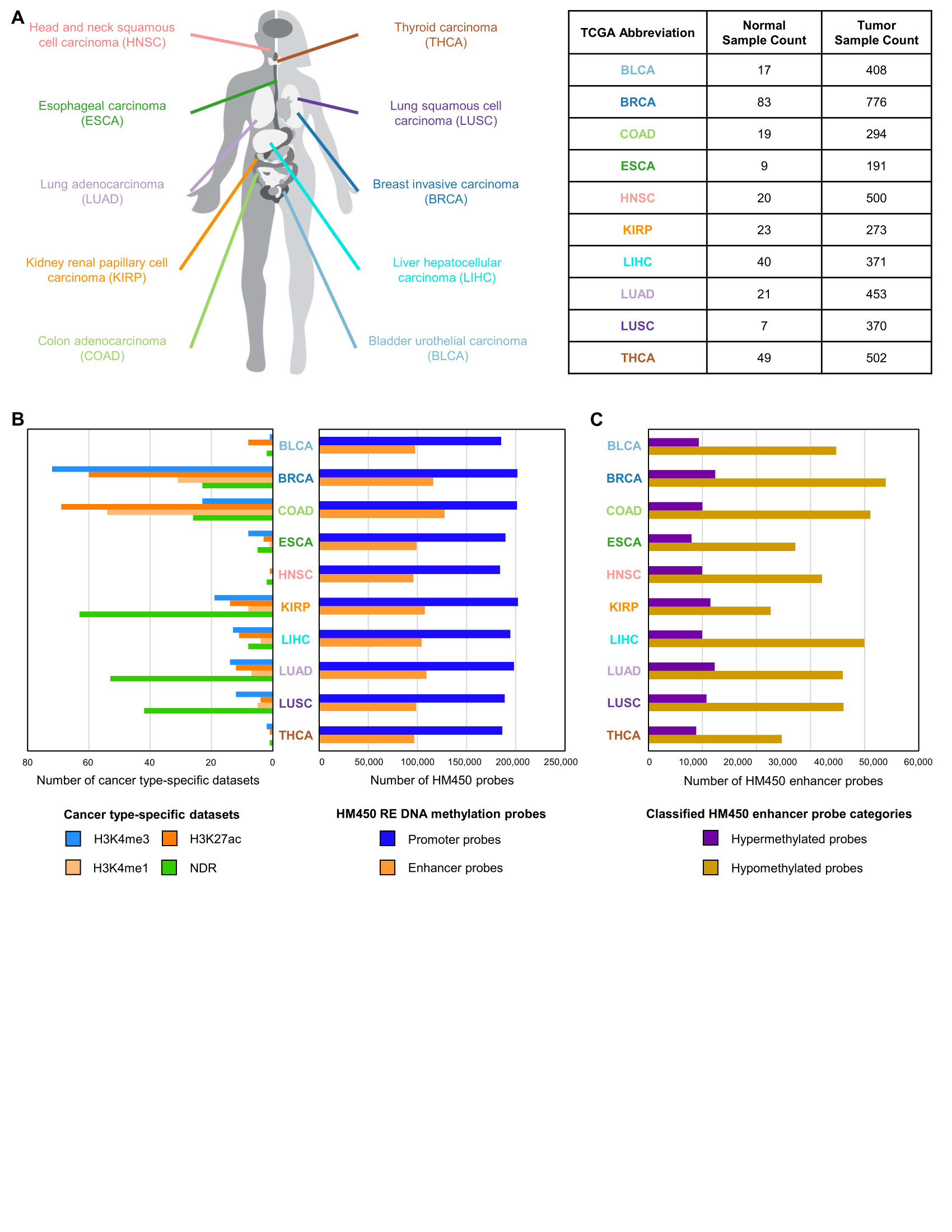
**

**Supplementary Figure S5. Application of TENET to pan-cancer data and identification of dysregulated REs using TCGA data**

(A) Diagram of the ten cancer types included in this study with their respective abbreviations and color coding (left) and table of the number of normal (control) and tumor (case) samples used for each cancer type in the study (right). (B) Bar plots displaying the number of cancer type-specific epigenomic datasets we included for each cancer type on the left, and the number of RE DNA methylation sites (RE sites, HM450 probes marking REs) identified per cancer type on the right. While all cancer types shared the same set of RE sites identified by the “consensus” and “ENCODE cCRE” epigenomic datasets, each cancer type also included a variable number of RE sites (HM450 probes) found using the cancer type-specific datasets from the pan-cancer dataset, including a relatively small number which are unique to each cancer type. (C) Bar plot of the total number of identified hypermethylated and hypomethylated enhancer sites (HM450 probes) in the ten cancer types.

**
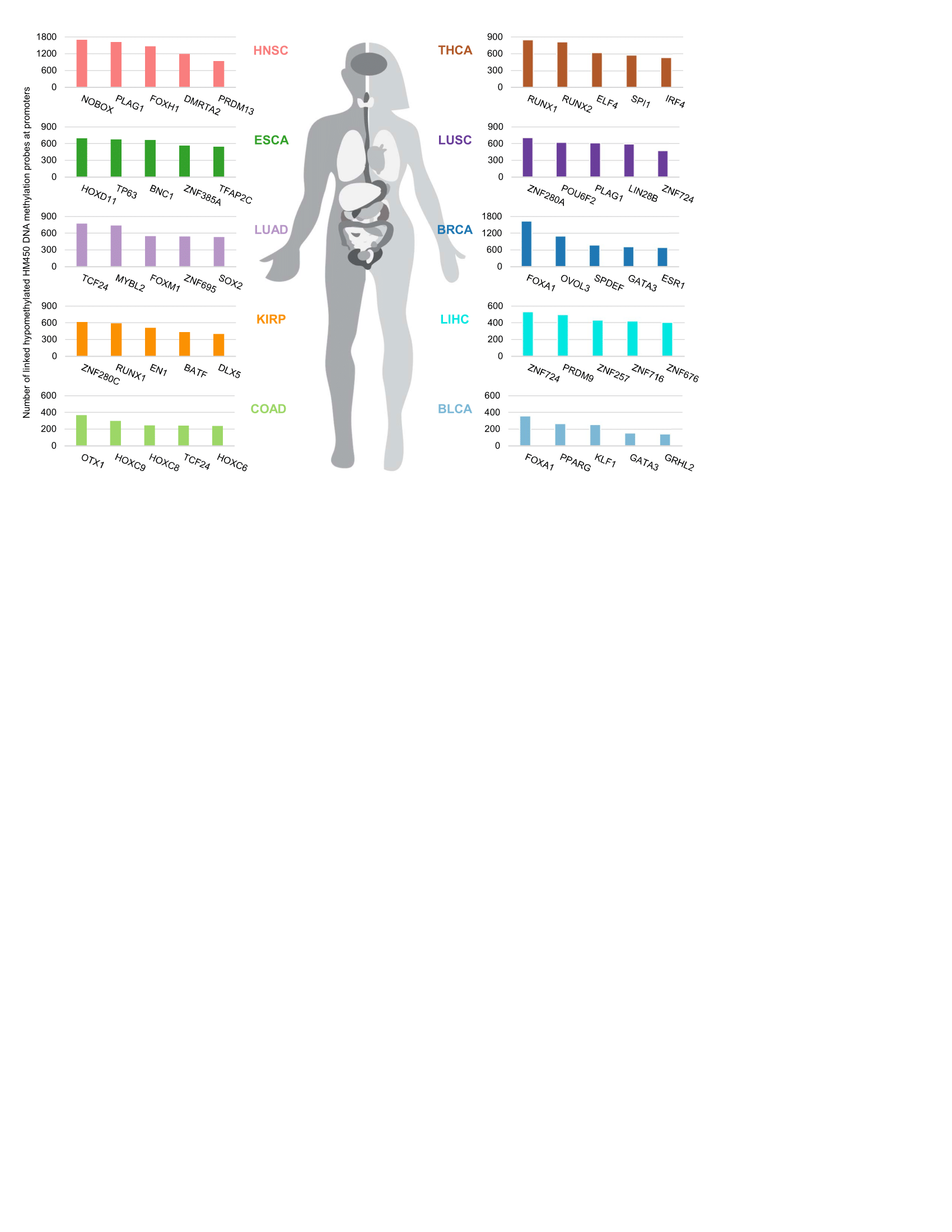
Supplementary Figure S6. Identification of key TFs linked to promoter sites using pan-cancer data**

Bar plots displaying the top 5 TFs linked to the largest number of hypomethylated promoter sites (HM450 probes) are shown for each cancer type.

**Supplementary Figure S7. Downstream analyses of key TFs and linked enhancer sites using TENET step 7 functions**

(A) Plots show the survival proportion over time of patients from the TCGA KIRP cohort with the 4^th^ (highest), 3^rd^, 2^nd^, and 1^st^ (lowest) quartiles of the expression of *MYBL2*, which is the most strongly survival-associated key TF identified through TENET KIRP analysis. (B) Plots show the survival proportion over time of patients from the TCGA KIRP cohort with the 4^th^ (highest), 3^rd^, 2^nd^, and 1^st^ (lowest) quartiles of the DNA methylation of cg24257495, which is one of the most strongly survival-associated enhancer sites (HM450 probes) identified through TENET KIRP analysis. (C) The enhancer site cg12002139 was found to be linked to RUNX1 in the KIRP dataset through TENET KIRP analysis, and this site is in the vicinity of RUNX1’s binding motif and within a RUNX1 ChIP-seq peak in HEK293T cells. The bottom track shows motifs of other transcription factors that are predicted to bind near cg12002139. (D) The potential target genes of enhancer site cg02911387 identified through TENET LIHC analysis were determined by integrating topologically associating domain (TAD) information obtained from a HepG2 cell line Hi-C dataset.
